# Miscellany

**Published:** 1903-08

**Authors:** 


### Miscellany.

Adrenalin in Devitalization of Pulps.—In an article on Adrenalin, in the June *Items of Interest,* W. Clyde Davis, M.D., D.D.S., of Lincoln, Neb., claims that he can painlessly extirpate the pulp in from one to three minutes. His method is as follows:

"Apply dam, if possible, and dry cavity. If pulp is not exposed, but covered by a layer of softened dentine, apply first a drop of adrenalin, then one drop of a forty per cent. solution of formalin. If quite a distance from the pulp, use slight but continued pressure with a rubber plug for a few seconds. You can now excavate to complete or near exposure painlessly. You are now where we all usually make our application of arsenic for devitalization, and are ready to begin with the operation."

He now applies one drop of adrenalin, lays in the cavity a few crystals of cocaine, or a one-sixth grain soluble tablet; applies one drop of a forty per cent. solution of formalin, and exerts gentle pressure with a rubber plug, increasing pressure as no pain is experienced by patient, and in from forty to sixty seconds is able to uncover the pulp-chamber and pass broach along the side of the pulp-chamber to the apex of the root.

In case any pain is felt, he repeats the application, not omitting the formaldehyde. After a root-canal dressing of camphophénique has remained twenty-four hours, he fills the canals.

The advantages claimed for this method are, first, it is painless; second, it saves time; third, the color of the tooth never changes; fourth, after-soreness is very slight, frequently wanting; fifth, the application is a powerful antiseptic. He claims that adrenalin alone is an obtunder of sensitive dentine.

---

The Manipulation and Care of Cements.—To obtain the best results with any dental cement, care is required in mixing together the liquid and the powder. They should be placed on the slab sufficiently far apart that they will not accidentally commingle. A very little of the powder should be first mixed with all the liquid intended to be used in the mix, and thoroughly spatulated. This is an important point. While more powder may be added after mixing has been commenced, it is always detrimental to add any more of the liquid. The first portions of powder mixed with the liquid changes its chemical character and prepares it for properly uniting with the rest. Therefore, it becomes important that the first portion should be thoroughly mixed and incorporated with all the liquid intended to be used. Further additions of the powder should be made a little at a time, each addition being thoroughly spatulated. Especially is this important in summer time; it will make all the difference between smooth plasticity with desirable setting qualities and a granular consistence with objectionably quick setting. The usual method of *slopping* the liquid and the powder together is detrimental in all cases.

In order to prevent undesirable change in the liquid it should not be used from the bottle in which it comes, but a portion should be transferred to a bottle having a telescoping glass cap instead of a cork fitting within the neck. The S. S. White No. 6 office preparation bottle is, perhaps, the best obtainable. With the ordinary container, with ordinary handling, there will always be more or less of the liquid standing about the cork, exposed to the air, which becomes changed in character, and on each removal of the cork contaminates that contained in the bottle.

Platino-iridium is the best metal for the spatula, and if socketed into some other metal need not be especially expensive. Next to this, a high grade of German silver, the so-called "platinoid," may be used, or where a slight modification of color is not objectionable, coin silver or an alloy of silver and copper in proper proportions to give the maximum rigidity and hardness, would be beneficial from a chemical stand-point, supposing that a slight abrasion and chemical action of the metal should occur. Phosphates of these metals have a very salutary effect, whereas phosphate of iron, which is necessarily formed to a certain extent when a steel spatula is used, is very detrimental.

Cleanliness in every procedure in connection with a mix of cement is especially called for. An immaculately clean spatula and slab should be used for every mix.—DR. W. V. B. AMES, *Dental Cosmos.*

---

EXTRACTION OF CANINES.—Dr. L. P. Haskell, in the *Dental Review,* advises the extraction of canines in upper jaw when they are the only teeth remaining in that jaw.

---

SENSITIVE NECKS OF TEETH.—Apply saturated solution of carbonate of potassium in glycerin. Repeat at intervals until sensitiveness is relieved.—*Dental Review.*

